# Investigation of Swallowing Performance and Family Impact in Children With Speech and Language Delay

**DOI:** 10.1111/1460-6984.70247

**Published:** 2026-04-13

**Authors:** Fahri Koca, Serkan Bengisu, Numan Demir

**Affiliations:** ^1^ Department of Speech and Language Therapy, Faculty of Health Sciences Istanbul Atlas University Istanbul Turkey; ^2^ Hacettepe University Faculty of Physical Therapy and Rehabilitation Ankara Turkey

**Keywords:** family quality of life, feeding and swallowing difficulties, feeding performance, oral motor functions, speech and language disorders, swallowing disorders

## Abstract

**Objective:**

This study aims to examine the relationship between delayed speech, feeding, and swallowing performance in children with speech and language delay (SLD) by comparing their feeding and swallowing performance with typically developing children, and to assess the impact of swallowing performance on families.

**Methods:**

A total of 60 children aged 24–60 months whose mother language was Turkish and their families participated in the study. Participants were divided into two groups: 40 children diagnosed with SLD and 20 typically developing children. Swallowing performance were assessed using the Pediatric Eating Assessment Tool (PEDI EAT‐10), the Oral Speech Mechanism Screening Examination‐Third Edition (OSMSE‐3), and the Karaduman Chewing Performance Scale (KCPS). Nutritional status was assessed using the Functional Oral Intake Scale (FOIS), while family impact was measured using the Pediatric Quality of Life Inventory (PedsQL) and the Feeding/Swallowing Impact Survey (FS‐IS).

**Results:**

Results showed significant differences between the two groups in PEDI‐EAT‐10, OSMSE‐3, and KCPS scores. The SLD group demonstrated significantly higher PEDI‐EAT‐10 scores (1.60 ± 4.21) compared with the typically developing control (TDC) group (0.00 ± 0.00; *p* = 0.011). Similarly, KCPS scores were significantly elevated in the SLD group (0.60 ± 0.59) relative to the TDC group (0.05 ± 0.22; *p* = 0.001). In contrast, the total OSMSE‐3 score was significantly lower in the SLD group (40.40 ± 7.49) than in the TDC group (48.80 ± 2.07; *p* < 0.001), indicating poorer oral sensorimotor function among children with SLD. However, no significant difference was found in FOIS scores (*p* = 0.058). Regarding family impact, significant differences were observed in PedsQL and FS‐IS scores (*p* = 0.001; *p* = 0.044).

**Conclusion:**

In conclusion, the swallowing performance and oral motor function of children with SLD were found to be significantly different from children without SLD. This suggests a possible association between motor planning deficits and the co‐occurrence of speech and language disorders and swallowing dysfunction. In addition, families of children with SLD reported lower quality of life and greater concerns about feeding and swallowing. These findings underscore the importance of addressing both SLD and swallowing disorder concurrently in clinical practice.

**WHAT THIS PAPER ADDS:**

*What is already known on this subject*

- Speech and language delay (SLD) is among the most common developmental disorders in early childhood and often co‐occurs with feeding and swallowing difficulties. Both speech and swallowing rely on shared neuromuscular and motor planning processes, suggesting a potential link between the two. Previous studies indicate that early feeding/swallowing challenges may serve as risk indicators for later language impairments. However, existing research has primarily focused on communication and learning outcomes, with limited attention given to swallowing performance in children with isolated SLD and the psychosocial impact on their families.

*What this paper adds to the existing knowledge*

- This study demonstrates that children with SLD exhibit significantly poorer swallowing performance and oral motor function compared to typically developing peers, despite showing no clinically significant nutritional limitations. It also highlights that families of children with SLD experience lower quality of life and greater daily challenges related to feeding and swallowing. By excluding confounding conditions such as neurological disorders and autism, this study provides clearer evidence of the association between SLD and swallowing dysfunction, as well as its negative impact on families—an area that has been largely underexplored in the literature.

*What are the potential or actual clinical implications of this study?*

- The findings underscore the need for clinicians to assess swallowing and oral motor skills in children presenting with SLD, even when overt feeding problems are not reported. Early identification of swallowing difficulties can support timely interventions that may mitigate developmental challenges. Moreover, given the demonstrated impact on families, clinical management should incorporate caregiver support and family‐centered approaches. Integrating swallowing assessments into routine SLD evaluations could improve diagnostic accuracy, guide multidisciplinary interventions, and ultimately enhance both child outcomes and family quality of life.

## 1 Introduction

Speech and language delay (SLD) refers to a delay in the acquisition of speech and/or language skills relative to age‐related developmental expectations (Sunderajan and Kanhere 2019). In the present study, SLD is operationally defined as a clinically identified delay in expressive and/or receptive language development based on standardized speech–language assessments and clinical judgment, in children who do not meet diagnostic criteria for developmental language disorder (DLD), intellectual disability, hearing loss, autism spectrum disorder, or neurological disease. In contrast to DLD, which is characterized by persistent and pervasive language impairment with a presumed neurodevelopmental basis, SLD is considered a broader and often earlier‐identified developmental delay that may resolve or evolve over time (Archibald 2024). This distinction is clinically important, as children with SLD often constitute a heterogeneous group whose developmental trajectories and associated functional difficulties may vary substantially over time. SLD is a common condition observed in children, with prevalence estimates suggesting that a substantial proportion of preschool‐aged children are affected during critical periods of language acquisition (Berkman et al. 2015; Feltner et al. 2024).

Speech and language development is closely linked to oral–motor control, as both speech production and swallowing rely on coordinated neuromuscular activity of the oral and facial musculature. Given this shared motor foundation, children with SLD may demonstrate delays not only in communication but also in feeding and swallowing functions, potentially increasing the risk of dysphagia‐related difficulties (Papadopoulou et al. 2024). Previous studies have reported that some children with SLD experience swallowing problems (Arvedson et al. 2019; Delaney and Arvedson 2008), and feeding and swallowing difficulties observed in infancy may serve as early indicators of later SLDs (Şahan et al. 2020).

Lefton‐Greif and Arvedson (2007) suggested that feeding and swallowing difficulties may influence early speech and language development and could predict subsequent language delays (Lefton‐Greif and Arvedson 2007). Similarly, Kallambettu et al. (2017) reported that early feeding and swallowing difficulties may affect later speech and language outcomes, indicating that feeding difficulties could represent early signs of SLD. Malas et al. (2015) further demonstrated that feeding and swallowing difficulties were significantly more prevalent in children with co‐occurring language and motor disorders compared to those with isolated language disorders, reinforcing the association between feeding–swallowing functions and speech–language development (Malas et al. 2015).

Feeding and swallowing are integral components of children's daily functioning, particularly during early childhood, when parents assume a primary caregiving role during mealtimes. Successful feeding and adequate nutritional intake are often perceived as indicators of parental competence (Shilts et al. 2021). Consequently, feeding and swallowing difficulties may increase caregiver burden and parental stress, adversely affecting family well‐being (Hewetson and Singh 2009). Parents of children with feeding difficulties frequently report heightened anxiety, disruptions to family routines, and reduced quality of life, particularly when feeding problems coexist with developmental delays (Gent et al. 2025; Blennerhassett et al. 2025). Thus, swallowing difficulties may exert a dual impact by affecting both child functioning and family dynamics (Alnıaçık and Bengisu 2025).

SLD is among the most prevalent developmental conditions negatively affecting communication skills and quality of life and often remains clinically unrecognized until later childhood. These delays are frequently identified between 3 and 5 years of age, underscoring the importance of early risk indicators that may adversely influence language development. The literature emphasizes the need for early markers to identify children at risk for speech and language disorders, and swallowing and feeding difficulties have been proposed as potential indicators that may interfere with language acquisition (Malas et al. 2017). However, empirical studies specifically examining the relationship between SLD and feeding–swallowing performance remain limited.

Moreover, while existing research has largely focused on the effects of speech and language disorders on children's learning and behavioral outcomes, the impact of feeding and swallowing difficulties on family quality of life has received comparatively little attention (van der Agt et al. 2005). Although swallowing disorders are recognized as factors that negatively affect caregiver quality of life, most studies have examined medically complex or neurologically impaired populations, and comprehensive investigations addressing caregiver experiences in families of children with SLD are scarce (Yi et al. 2024). Understanding family impact is particularly important in SLD, as these children often appear physically healthy, which may contribute to under‐recognition of caregiver stress and unmet support needs.

Therefore, the primary objectives of this study were to compare children with SLD and typically developing peers in terms of feeding and swallowing performance and to evaluate the impact of swallowing difficulties on family functioning. By examining both child‐level swallowing performance and family‐level outcomes, this study aims to provide a more comprehensive understanding of the functional and psychosocial implications of SLD.We hypothesized that children with SLD would exhibit poorer swallowing performance and that their families would report a greater negative impact compared to families of typically developing children.

## 2 Method

### 2.1 Participants

This study was conducted between January 1 and 30 June 2024, with a total of 60 children aged 24–60 months, all of whom were native Turkish speakers. The participants were divided into two groups. The study group consisted of 40 children with delayed speech and language development (SLD) and their families, while the control group consisted of 20 typically developing children and their families.

Inclusion criteria for the study group were as follows: (i) individuals whose native language is Turkish, (ii) individuals diagnosed with “speech and language delay” as documented in the Guidance and Research Center (RAM) report, and (iii) individuals with language disorders affecting more than one component of language (phonology, morphology, syntax, semantics, and pragmatics). The exclusion criteria for the study group were as follows: (i) children with significant sensory impairments such as hearing or vision loss, (ii) children with a genetic disorder in addition to delayed speech and language development, (iii) children diagnosed with neurological conditions (e.g., cerebral palsy), autism spectrum disorders, fluency disorders, syndromes, or motor speech disorders, and (iv) children with oral or craniofacial anomalies. In addition, children born preterm were excluded from the study because of the well‐established association in the literature between preterm birth and speech and language disorders. The inclusion criteria for the control group were as follows: voluntary participation in the study, typically developing, no diagnosed developmental delay, and no history of physiological or neurodevelopmental disorders. The adequacy of the sample size was evaluated using G*Power version 3.1. Assuming a medium effect size (*r* = 0.30), a significance level of 5% (*α* = 0.05), and a statistical power of 80% (1−*β* = 0.80), the a priori power analysis indicated that a minimum of 54 participants was required for between‐group comparisons. The inclusion of a total of 60 participants in the present study therefore provided sufficient statistical power for the planned analyses.

The diagnosis of SLD was based on the RAM (Guidance and Research Center) report. In Turkey, Disability Health Board reports are issued to support the education of children with special needs. These reports serve as a basic tool for determining the amount and type of special education and rehabilitation services needed by children. These reports are prepared by a medical committee in a fully equipped state hospital, consisting of qualified specialists such as child and adolescent psychiatrists and pediatricians. After the report is prepared, the RAM conducts the evaluation process and develops individualized educational plans for the child. The RAM assessment examines the child's language skills in detail using both standardized and non‐standardized methods. Non‐standardized assessment methods include analysis of speech samples, evaluation of receptive and expressive language skills, and observation of language skills in various communicative contexts such as structured, semi‐structured, and free‐play settings. These methods are used to gain a comprehensive understanding of the child's language development and communication skills. Standardized assessment tools used in the evaluation include the Turkish versions of the Denver Developmental Screening Test II (Anlar et al. 2009) and the Peabody Picture Vocabulary Test (Dunn and Dunn 1997), which measure children's language and developmental skills.

The study was approved by the Ethics Committee for Non‐Interventional Clinical Research of Istanbul Atlas University and conducted in accordance with the Declaration of Helsinki (Date: 19.01.2024, No: 37158). All participants fulfilled the stated inclusion criteria.

### 2.2 Assessment Tools and Implementation Procedure

In this study, participants were divided into two groups: children in the SLD group and their primary caregivers were selected from the same special education and rehabilitation center, while children in the control group and their caregivers were selected from a public school in the same region. The data collection process was carried out through face‐to‐face interviews. Participants within each group were selected from the same educational institution, and all participants came from similar socioeconomic and cultural backgrounds. Children in the SLD group had been diagnosed with SLD based on RAM reports, whereas children in the control group were selected from among those whose teachers and parents reported no developmental or language difficulties. Interviews took place individually in a quiet, private room free from distracting stimuli. Each session lasted approximately 45 min. Prior to participation, all caregivers were informed about the study's purpose and scope, and written informed consent was obtained. The assessment environment and procedures were carefully structured to maximize the validity and reliability of the data collected.

Swallowing performance in the participating children were evaluated using the Pediatric Eating Assessment Tool (PEDI‐EAT‐10), the Oral Speech Mechanism Screening Examination—Third Edition (OSMSE‐3), and the Karaduman Chewing Performance Scale (KCPS). Nutritional status was assessed using the Functional Oral Intake Scale (FOIS). To determine the impact of SLD and feeding/swallowing performance on families, the Pediatric Quality of Life Inventory (PedsQL) and the Feeding/Swallowing Impact Survey (FS‐IS) were administered.

The assessments were administered in a fixed order to ensure procedural consistency and to minimize child fatigue. The data collection session began with the administration of the PEDI‐EAT‐10, FS‐IS, and PedsQL questionnaires to caregivers, each taking approximately 10 min. Subsequently, the researcher conducted the OSMSE‐3 and FOIS assessments with the child (approximately 10–15 min in total). Finally, the KCPS was administered (approximately 5 min). To reduce the potential impact of fatigue, short breaks were provided when necessary, and assessments were discontinued or rescheduled if the child showed signs of distress, inattention, or non‐cooperation.

The questionnaires (PEDI‐EAT‐10, PedsQL, and FS‐IS) were administered in a clinical setting by the researcher. To minimize misinterpretation due to low educational level or visual impairments, the researcher read all questionnaire items and response options aloud to the participants. This approach aimed to enhance clarity and reduce the risk of inaccurate or misleading responses. The questionnaires were administered uniformly to all participants to ensure procedural consistency and eliminate bias during data collection.

The FOIS and OSMSE‐3 assessments were completed directly by the researcher. For the KCPS assessment, children were asked to choose one type of biscuit (plain, cocoa, or chocolate cream‐filled) in a classroom setting. A video recording focusing only on the mouth region was taken during the chewing process. Although each child received only one biscuit, recordings were repeated if technical issues occurred or if the child moved away from the camera during filming. All KCPS recordings were independently analyzed and scored by two independent speech and language therapists. Inter‐rater reliability for the KCPS was calculated using intraclass correlation coefficients (ICC), which demonstrated excellent agreement between raters (ICC > 0.90).

#### 2.2.1 Swallowing Function Assessments

##### 2.2.1.1 Functional Oral Intake Scale (FOIS)

The nutritional level of the children was determined using the FOIS. This scale is a 7‐point ordinal scale that assesses functional oral intake levels. The FOIS allows the assessment of the patient's daily oral intake at different levels. The scale includes different levels of non‐oral (tube‐fed) feeding between levels 1 and 3, while levels 4–7 include oral feeding levels (Crary et al. 2005).

##### 2.2.1.2 Pediatric Eating Assessment Tools (PEDI‐EAT‐10)

The PEDI‐EAT‐10 is the pediatric version of the EAT‐10 scale, which measures the severity of dysphagia symptoms in adults, and was developed by Arslan et al. (Serel Arslan et al. 2018a). This parent‐reported tool is able to detect the risk of penetration/aspiration in children, and helps to identify pediatric patients at risk for dysphagia‐related complications. The test consists of 10 questions. A score of 0 indicates “no problem” and a score of 4 indicates “severe problem” for each question. A PEDI‐EAT‐10 score of 4 or higher indicates a risk of swallowing disorders, with higher scores reflecting more severe dysphagia symptoms.

##### 2.2.1.3 Oral Speech Mechanism Screening Examination (OSMSE‐3)

Children's oral structures and motor skills were assessed using the Oral Speech Mechanism Screening Examination‐Third Edition (OSMSE‐3). The OSMSE‐3 is a standardized assessment tool designed to evaluate anatomical structures and physiological functions that may be associated with speech or language disorders. The test examines oral structures and functions in two phases.

**Level 1** assesses anatomical structures, including the lips, tongue, jaw, teeth, hard palate, soft palate, pharynx, breathing, and velopharyngeal mechanism.

**Level 2** evaluates oral functions in two subcategories: non‐speech motor functions and speech‐related motor functions. Non‐speech motor functions include rounding of the lips, retraction of the corners of the mouth, inflation of the cheeks, biting the lower lip, and moving the tongue up, down, sideways, and toward the hard palate. Speech‐related oral functions are assessed using the diadochokinesis test, which involves rapid repetition of syllable sequences (e.g., pa‐ta‐ka). The test typically takes between 5 and 10 min to administer (Robbins and Klee 1987).

##### 2.2.1.4 Karaduman Chewing Performance Scale (KCPS)

The Karaduman Chewing Performance Scale (KCPS), a valid and reliable tool for assessing chewing performance levels in children, was used in this study. The KCPS consists of five levels ranging from level 0 (“chewing within functional limits”) to level 4 (“no biting or chewing”). Children were instructed to bite and chew a standardized cookie, and their chewing behavior was observed to determine the appropriate level of performance (Serel Arslan et al. 2016).

#### 2.2.2 Family Impact Assessment

##### 2.2.2.1 Pediatric Quality of Life Inventory (PedsQL)

The Family Impact Module of the Pediatric Quality of Life Inventory (PedsQL) is a parent‐reported measure developed to assess the impact of pediatric chronic health conditions on parents and families. The scale consists of six subscales that measure parent‐reported functioning: Physical Functioning (6 items), Emotional Functioning (5 items), Social Functioning (4 items), Cognitive Functioning (5 items), Communication (3 items), and Worry (5 items). In addition, there are two subscales that assess family functioning: Daily Activities (3 items) and Family Relationships (5 items). The scale uses a five‐point Likert response format with the following options: Never, Almost Never, Sometimes, Often, and Almost Always, with scores of 100, 75, 50, 25, and 0, respectively. Higher scores indicate better functioning (i.e., fewer negative effects). The PedsQL Family Impact Module Total Scale Score was calculated by summing the scores of the 36 items and dividing by the number of items answered (Varni et al. 1998).

##### 2.2.2.2 The Feeding/Swallowing Impact Survey (FS‐IS)

The FS‐IS is a parent‐report instrument designed to assess the impact of children's feeding and swallowing difficulties on their caregivers. It was developed by Lefton‐Greif et al. in 2014 and consists of 18 items grouped into three subdomains: Daily Activities, Worries, and Feeding Difficulties. Each item is scored on a 5‐point scale, with 1 indicating never and 5 indicating almost always. Higher scores indicate a lower quality of life. The survey was adapted into Turkish, and its validity and reliability were established by Serel Arslan et al. (2018b).

##### 2.2.2.3 Statistical Analysis

Statistical analysis of the data was performed using SPSS 25.0 (SPSS Inc., Chicago, IL, USA). The Shapiro–Wilk test was used to assess whether the data were normally distributed. Descriptive statistics included mean, standard deviation (mean ± SD), minimum and maximum values, frequencies, and percentages. The non‐parametric Mann–Whitney *U* test was used because the data deviated significantly from a normal distribution. In the analyses, *p* < 0.05 was accepted as a statistically significant level. To control for the potential confounding effect of age differences between the groups, analysis of covariance (ANCOVA) was additionally performed for the primary swallowing and oral motor outcome variables, with age included as a covariate in the model. Given the deviation of the data from a normal distribution, non‐parametric methods were used for the primary analyses, whereas ANCOVA was conducted as a supplementary analysis to specifically evaluate the effect of age on the main outcomes.

Because non‐parametric tests were used for between‐group comparisons, effect sizes were calculated based on the Mann–Whitney U test using the r coefficient (*r* = Z/√N). Effect size values were interpreted as small (*r* = 0.10), medium (*r* = 0.30), and large (*r* = 0.50). For age‐adjusted analyses, effect sizes were reported using partial eta squared (partial *η* ^2^). Ninety‐five percent confidence intervals (95% CIs) for effect sizes were estimated using bootstrap methods for non‐parametric variables and model‐based confidence intervals for parametric analyses.

## 3 Results

The mean age of the children diagnosed with delayed SLD was 4.37 ± 0.7 years, while the mean age of the typically developing control group was 3.60 ± 0.5 years. In the SLD group, 12.5% (five children) were 3 years old, 37.5% (15 children) were 4 years old, and 50% (20 children) were 5 years old. In the typically developing control group, 40% (eight children) were 3 years old, 60% (12 children) were 4 years old, and no participants were 5 years old. There was no statistically significant difference in age or gender between the groups, so the groups are homogeneously distributed. The demographic and clinical characteristics of the participants are shown in Table 1.

**TABLE 1:** Demographic information of the participants.

		SLD (*N* = 40)		TDC (*N* = 20)		
		Participants	Mean ± SD	Participants	Mean ± SD	*p*
Age (years)	**3**	5 (12.5%)	4.37±0.7	8 (40%)	3.60±0.5	0.065
	**4**	15 (37,5%)		12 (60%)		
	**5**	20 (50%)		0 (0%)		
Gender	**Female**	12 (30%)		6 (30%)		0.977
	**Male**	28 (70%)		14 (70%)		

*Note*: Mann–Whitney *U* test, ^*^ *p* < 0.05.

Abbreviations: SD: standard deviation; SLD, speech and language delay; TDC, typically developing control.

### 3.1 Results of Swallowing and Feeding Assessment Tools

The PEDI EAT‐10, KCPS, and OSMSE‐3 were used to assess participants' swallowing function, while the FOIS was used to assess their feeding status (Table 2).

**TABLE 2:** Swallowing assessment results of the participants.

	SLD (*n* = 40)			TDC (*n* = 20)				
	Min	Max	Mean ± SD	Min	Max	Mean ± SD	U	*p*
PEDI EAT‐10	0	23	1.60 ± 4.21	0	0	0.00 ± 0.00	290.0	0.011^*^
KCPS	0	2	0.6 ± 0.59	0	1	0.05 ± 0.22	199.0	0.001^*^
OSMSE‐3								
‐Structure score	19	31	25.13 ± 3.44	28	30	28.75 ± 0.79	649.5	0.001^*^
‐Function score	8	24	15.38 ± 4.45	18	22	19.60 ± 1.39	622.0	0.001^*^
‐Total score	28	55	40.40 ± 7.49	46	52	48.80 ± 2.07	648.5	0.001^*^
FOIS	6	7	6.83 ± 0.38	7	7	7.00 ± 0.00	470.0	0.058

*Note*: Mann–Whitney *U* test, ^*^ *p* < 0.05.

Abbreviations: Max, maximum; Min, minimum; SD, standard deviation; SLD, speech and language delay; TDC, typically developing control.

In the SLD group, the mean PEDI EAT‐10 and KCPS scores were 1.60 ± 4.21 and 0.6 ± 0.59, respectively, whereas in the typically developing control (TDC) group, the mean PEDI EAT‐10 and KCPS scores were 0.00 ± 0.00 and 0.05 ± 0.22, respectively. A statistically significant difference was found between the two groups in PEDI EAT‐10 and KCPS scores (*p* = 0.011, *p* = 0.001). Inter‐rater reliability analysis of the KCPS scores demonstrated excellent agreement between the two independent raters. The mean KCPS scores were 0.42 ± 0.39 for rater 1 and 0.41 ± 0.37 for rater 2, with an intraclass correlation coefficient of ICC = 0.95 (95% confidence interval: 0.96–0.99). According to the KCPS results, in the SLD group (*n* = 40), 45% (18 children) scored 0, 50% (20 children) scored 1, and 5% (2 children) scored 2. In the TDC group (*n* = 20), 95% (19 children) scored 0, while only 5% (1 child) scored 1. To control for the potential confounding effect of age, ANCOVA was performed with age included as a covariate. In this model, the group effect for PEDI EAT‐10 scores was no longer statistically significant (F(1,57) = 0.93, *p* = 0.338, partial *η* ^2^ = 0.016), and the effect of age on PEDI EAT‐10 scores was also not significant (*p* > 0.05).

According to the OSMSE‐3, the mean structure, function, and total scores in the SLD group were 25.13 ± 3.44, 15.38 ± 4.45, and 40.40 ± 7.49, respectively. In the TDC group, the mean structure, function, and total scores were 28.75 ± 0.79, 19.60 ± 1.39, and 48.80 ± 2.07, respectively. There was a statistically significant difference in groups in OSMSE‐3 scores between two groups (*p* = 0.001). ANCOVA analysis controlling for age demonstrated that the group effect on the OSMSE‐3 total score remained statistically significant (F(1,57) = 14.55, *p* < 0.001), with a large effect size (partial *η* ^2^ = 0.203). The contribution of age to the model was not statistically significant (*p* > 0.05), indicating that the observed group difference was independent of age.

Based on the FOIS, the mean FOIS score was 6.83 ± 0.38 in the SLD group and 7.00 ± 0.00 in the TDC group. There was no statistically significant difference in FOIS scores between the two groups (*p* = 0.058). When age was included as a covariate in the ANCOVA model, the group effect for FOIS scores remained non‐significant (F(1,57) = 1.72, *p* = 0.195, partial *η* ^2^ = 0.029), and age did not significantly influence FOIS scores (*p* > 0.05). Accordingly, FOIS findings were interpreted cautiously, without attributing the absence of group differences to functional compensation mechanisms. Due to the ordinal structure of the KCPS, Pediatric Quality of Life Inventory (PedsQL), and Feeding/Swallowing Impact Survey (FS‐IS), as well as their limited category ranges, ceiling effects, or violations of normality and homogeneity of variance assumptions, age‐adjusted parametric ANCOVA analyses were not conducted for these variables. When effect sizes derived from between‐group comparisons were examined, group differences in OSMSE‐3 structure, function, and total scores demonstrated medium‐to‐large to large effect sizes (*r* ≈ 0.50–0.55; 95% confidence interval [CI] ≈ 0.27–0.71). The group difference in PEDI EAT‐10 scores demonstrated a medium effect size (*r* ≈ 0.30; 95% CI ≈ 0.12–0.48), whereas the observed difference in FOIS scores corresponded to a small effect size (*r* ≈ 0.25; 95% CI ≈ 0.01–0.46). In contrast, a large effect size was identified for the KCPS (*r* > 0.50; 95% CI ≈ 0.36–0.69).

### 3.2 Results of Family Impact Assessment Tools

The PedsQL was used to assess the impact on the participants' families, while the Feeding/Swallowing Impact Survey (FS‐IS) was used to assess the impact of feeding and swallowing difficulties on the families. The results of the PedsQL are shown in Table 3 and the results of the FS‐IS are shown in Table 4.

**TABLE 3:** Results of the participants obtained from PedsQL.

	*N*	Min	Max	Mean ± SD	*U*	*p*
SLD	40	0	81	46.5 ± 21.93	6.2	0.001^*^
TDC	20	0	9	96.4 ± 29.72		

*Note*: Mann–Whitney *U* test, ^*^ *p* < 0.05.

Abbreviations: Max, maximum; Min, minimum; SD, standard deviation; SLD, speech and language delay; TDC. typically developing control.

**TABLE 4:** Results of the participants obtained from FS‐IS.

FS‐IS		*N*	Min	Max	Mean ± SD	*U*	*p*
Daily activities	SLD	40	5	18	7.73 ± 3.46	564.0	0.005^*^
	TDC	20	5	6	5.30 ± 0.47		
Worry	SLD	40	7	27	9.70 ± 4.72	508.0	0.063
	TDC	20	7	12	7.70 ± 1.38		
Feeding difficulties	SLD	40	6	21	7.35 ± 3.25	439.5	0.375
	TDC	20	6	8	6.20 ± 0.52		
Total	SLD	40	18	65	24.73 ± 10.03	523.0	0.044^*^
	TDC	20	18	26	19.20 ± 2.02		

*Note*: Mann–Whitney *U* test, ^*^ *p* < 0.05.

Abbreviations: Max, maximum; Min, minimum; SD, standard deviation; SLD, speech and language delay; TDC, typically developing control.

The mean PedsQL score for participants in the SLD group was 46.5 ± 21.93, while the mean score for the typically developing control (TDC) group was 96.4 ± 12.72. A statistically significant difference in PedsQL scores was found between the two groups (*p* = 0.001).

The mean scores obtained for the families of children in the SLD group in daily activities, worry, feeding difficulties, and FS‐IS total scores were 7.73 ± 3.46, 9.70 ± 4.72, 7.35 ± 3.25, and 24.73 ± 10.03, respectively. For the children of families in the TDC group, the mean scores for activities of daily living, anxiety, feeding difficulties, and FS‐IS total scores were 5.30 ± 0.47, 7.70 ± 1.38, 6.20 ± 0.52, and 19.20 ± 2.02, respectively. A statistically significant difference was found between the two groups in the FS‐IS Daily Activities subdomain and total scores (*p* = 0.005, *p* = 0.044). However, no statistically significant difference was found between the SLD and TDC groups in the worry and feeding difficulties subdomains of the FS‐IS survey (*p* = 0.063, *p* = 0.375). Effect size analysis revealed that the between‐group difference in PedsQL scores demonstrated a very large effect size (*r* > 0.60; 95% CI ≈ 0.45–0.77). For the FS‐IS, the group difference in the daily activities subdomain showed a medium effect size (*r* ≈ 0.35; 95% CI ≈ 0.17–0.52), while the total score exhibited a small‐to‐medium effect size (*r* ≈ 0.25; 95% CI ≈ 0.05–0.43).

## 4 Discussion

Speech and language disorders often become clinically evident at relatively later stages of development and, despite their well‐documented negative impact on communication and quality of life, may remain undiagnosed until 3–5 years of age. Identifying early indicators that may signal increased risk for SLD is therefore of critical importance. Feeding and swallowing difficulties have been proposed as one such potential indicator, as they may influence language acquisition indirectly by limiting opportunities for sensorimotor exploration and communicative interaction (Malas et al. 2017). Within this framework, the present study should be considered exploratory in nature, aiming to contribute preliminary evidence regarding the co‐occurrence of swallowing‐ and feeding‐related characteristics in children with SLD. Rather than merely documenting group differences, the present study sought to elucidate the nature and underlying significance of swallowing‐ and feeding‐related findings in children with SLD, situating these findings within a broader developmental and psychosocial context.

The findings indicate that children with SLD exhibit measurable weaknesses in oral motor structure and function compared with typically developing peers. The most pronounced group differences were evident in the OSMSE‐3 scores, suggesting that impairments in oral motor mechanisms and motor planning may co‐occur with language delays. Importantly, these findings extend beyond a simple description of group differences, as the persistence of a strong group effect on the OSMSE‐3 total score after controlling for age demonstrates that oral motor and swallowing‐related difficulties in children with SLD cannot be attributed solely to developmental age differences. However, given the relatively small sample size, these findings should be interpreted cautiously and not as evidence of a definitive or universal characteristic of all children with SLD. This supports the interpretation that these impairments may represent a stable characteristic associated with SLD rather than a transient maturational lag.

At the same time, the attenuation of group effects for PEDI EAT‐10 and FOIS scores after age adjustment suggests that these measures may be more sensitive to age‐related developmental variability. This pattern highlights a potential dissociation between underlying oral‐motor inefficiencies and functional feeding outcomes, particularly in children who are able to compensate during daily feeding activities. Similar dissociations between structural or motor findings and functional feeding outcomes have been reported in previous pediatric feeding studies (Lefton‐Greif and Arvedson 2007; Arvedson 2008). These scales also demonstrated limited score dispersion and ceiling effects, which may have reduced their ability to detect subtle group differences once age was statistically controlled. Accordingly, the findings underscore the methodological importance of selecting assessment tools that capture qualitative aspects of feeding performance rather than relying solely on global intake‐based measures.

Previous literature has proposed shared neurodevelopmental substrates for speech, language, and oral motor functions (Lai et al. 2001; Maas et al. 2008). However, given the absence of direct neuroimaging or neurophysiological data in the present study, neurobiological explanations should be interpreted cautiously. Rather than implying direct neural causality, the current findings are more appropriately understood as reflecting functional co‐occurrence of language and oral motor challenges, potentially mediated by overlapping developmental processes such as motor planning, coordination, and sensorimotor integration (Shum et al. 2011).

Beyond neurodevelopmental explanations, feeding and swallowing difficulties observed in children with SLD may also be influenced by behavioral and environmental mechanisms. Feeding behaviors are embedded within daily routines and are shaped by caregiver practices, feeding styles, cultural norms, and socioeconomic context. Variations in parental responsiveness, feeding expectations, and mealtime structure may affect both oral motor skill development and opportunities for communicative interaction. Accordingly, feeding difficulties in children with SLD may not be purely motoric in origin, but may also reflect learned behaviors, adaptive strategies, or limited exposure to developmentally challenging feeding experiences. This interpretation is consistent with previous literature indicating that feeding difficulties are not invariably accompanied by objective motor impairments. In this regard, Burr et al. (2021) reported that similar oral muscles and structures are involved in both sucking and speech, suggesting that early sucking habits may have potential implications for speech development. They further noted that delays in language development may be associated with early feeding and sucking behaviors; however, they emphasized that the relationship between speech sound development and feeding or swallowing skills remains inconsistent and unclear.

Malas et al. (2015) conducted a retrospective study to examine the relationship between feeding and swallowing difficulties and later DLDs (Malas et al. 2015). Their findings indicated that the prevalence of feeding‐swallowing difficulties in children with SLD was significantly higher than the estimated prevalence in the general population. In particular, children with motor impairments were more likely to have feeding transition difficulties and drooling control problems, suggesting that feeding‐swallowing difficulties may serve as an early indicator of language disorders. These results support the notion that feeding‐swallowing difficulties may function as an early risk marker rather than a direct causal factor.

In their study, Lefton‐Greif and Arvedson (2007) highlighted the increasing prevalence of pediatric feeding and swallowing difficulties and their impact on children's health and functional status (Lefton‐Greif and Arvedson 2007). They noted that early disruptions in language development may occur and that impairments in swallowing function may negatively affect both motor and language development. These findings suggest that feeding and swallowing difficulties are significant factors contributing to delays in language development.

Similarly, the study conducted by Adams‐Chapman et al. (2013) demonstrated an association between feeding difficulties and delays in language development in extremely preterm infants (Adams‐Chapman et al. 2013). The authors emphasized that early identification of feeding and swallowing problems in preterm infants could play a critical role in preventing their adverse effects on language development. Taken together, these findings highlight the dynamic interplay between feeding difficulties and language development and underscore the importance of early surveillance and intervention.

However, a common feature of these studies in the literature is that they include children with genetic disorders, autism, fluency disorders, cerebral palsy (CP), syndromic diagnoses, and motor speech disorders in addition to feeding and swallowing difficulties. The key feature of our study is that it focuses exclusively on children with SLDs who do not have neurological deficits. Participants were required to have feeding and swallowing difficulties in addition to the SLD without any accompanying genetic disorders. Children diagnosed with autism, fluency disorders, CP, syndromic conditions, or motor speech disorders were excluded from the study. In addition, the absence of significant sensory impairments was considered an inclusion criterion.

In the present study, swallowing‐related assessment scores differed significantly between the SLD and TDC groups, indicating that swallowing difficulties are more pronounced in children with SLD. However, no statistically significant group differences were observed in FOIS scores. This finding should not be interpreted as evidence that swallowing difficulties are absent or fully compensated, as the FOIS primarily reflects overall oral intake level and may lack sensitivity to subtle qualitative differences in feeding performance. The fact that FOIS scores in both groups were near the upper limit suggests that the observed oral‐motor impairments do not translate into clinically or nutritionally significant limitations in daily feeding and may therefore be considered subclinical in nature. Moreover, the absence of qualitative mealtime observations and detailed caregiver reports limits the extent to which functional compensation or underlying mechanisms can be inferred, representing an important methodological consideration. Taken together, these findings suggest that although swallowing difficulties are more evident as a symptom in children with SLD, the disorder does not appear to directly affect feeding habits or skills. Consistent with previous literature, this pattern supports the interpretation that feeding‐related difficulties in this population may have a stronger behavioral component and highlights that language development and motor skills are shaped not only by biological factors but also by behavioral and environmental interactions.

The relationship between feeding difficulties and language acquisition is not clearly defined in the literature. From a neurological perspective, it has been hypothesized that the pathways involved in language development may be related to feeding processes. However, it has been reported that individuals with feeding and language disorders do not always have motor impairments. Furthermore, the absence of motor impairments in these children suggests that feeding difficulties may be more behavioral in natüre (Burr et al. 2021).

Understanding the impact of speech and language disorders on the quality of life of children and their families is critical to improving the effectiveness of interventions in this area. Examining not only the impact of delayed speech and language on communication skills, but also the impact on physical health, such as feeding and swallowing difficulties, highlights the need for a holistic approach to these issues. Examining the psychosocial impact of these conditions on families provides an important basis for developing strategies to improve the quality of life for both the child and the family. Both SLD and feeding difficulties can lead to various complications over time that negatively affect the child's daily life, peer interactions, and communicative functioning within the family. As a result, these challenges can have a negative impact on the quality of life of both children and their families (Adams‐Chapman et al. 2013). Studies on the impact of speech and language disorders on the family have generally focused on the impact of speech and language problems on learning and behavioral aspects. However, the impact of swallowing and feeding disorders on the family has not been well studied (van der Agt et al. 2005). Another key issue addressed in this study is the impact of swallowing and feeding difficulties observed in individuals with SLD on their families. Swallowing disorder is considered to be a significant factor affecting the quality of life of caregivers. However, no research has addressed the swallowing concerns of caregivers of children with SLD. To the best of our knowledge, this is the first study to examine the impact of feeding and/or swallowing difficulties in children with SLD on their families. Based on our findings, the quality of life of caregivers of children with SLD was found to be lower than that of caregivers of children without SLD. In addition, the severity of the child's swallowing difficulties was found to have a significant impact on the family's quality of life. The results of our study suggest that feeding and/or swallowing difficulties significantly affect not only children with SLD, but also their families. However, no significant differences were found between the groups in the anxiety and feeding difficulty subscales of the FS‐IS. This finding may be attributable to factors such as caregiver adaptation to the condition, lower severity of symptoms, or cultural differences in perception.

A key contribution of this study lies in its examination of family impact. The significantly lower PedsQL scores in the SLD group underscore the broader psychosocial burden associated with SLD. Notably, the strong effect size observed for caregiver quality of life suggests that even subclinical swallowing or feeding concerns may meaningfully affect family well‐being. However, the limited sample size and the imbalance between groups in this study may have limited the statistical power to detect significant differences in variables such as caregiver burden and family quality of life. Further research is needed to explore the psychosocial impacts of swallowing difficulties on families and how these effects can be managed.

In light of these findings, it is concluded that the impact of SLD and swallowing disorder on the family should be addressed with a holistic approach. Considering that such disorders affect not only the quality of life of the child but also that of the family, the importance of family support programs and interventions must be emphasized. Future studies may contribute to the development of multidisciplinary approaches that meet the needs of both the child and the family.

These findings should be interpreted in light of several limitations of the study. The relatively small sample size and the imbalance in age distribution across groups represent important constraints on the generalizability of the results. In our country, a RAM report is considered sufficient for the diagnosis of SLD; therefore, the identification of individuals with SLD in this study was based solely on RAM reports, and no detailed, standardized language assessments were administered. Similarly, the control group was defined based on parental reports and health records indicating typical development, but no standardized developmental or language screening tools were applied to this group. As a result, the possibility of undetected or low‐level language delays among children in the control group cannot be entirely ruled out. In addition, the limited overall sample size and numerical imbalance between groups may have reduced the statistical power of the study. Future research should employ standardized and norm‐referenced assessments with higher diagnostic validity for SLD and adopt a comprehensive evaluation of expressive, receptive, and pragmatic language skills. Furthermore, systematic language screening of the control group, larger sample sizes, and improved numerical balance between groups would enhance the generalizability of future findings. Finally, longitudinal studies may provide stronger evidence regarding the causal relationships between swallowing or developmental feeding difficulties and language development.

## 5 Conclusion

In conclusion, significant differences were observed in the swallowing performance and oral motor structure–function assessments of children with SLD compared to children without SLD. These findings suggest that SLD may be associated with concomitant vulnerabilities in swallowing‐related motor processes; however, this relationship should be interpreted cautiously and considered exploratory, given the behavioral nature of the assessments and the absence of direct neurobiological measures. In addition, quality of life scores and feeding‐ and swallowing‐related impact scores were significantly higher in the families of children with SLD compared to typically developing controls, indicating that the consequences of SLD extend beyond the child to affect the broader family context. These results underscore the clinical relevance of incorporating caregiver‐reported outcomes into the assessment process, while acknowledging that family impact may also be shaped by contextual and sociocultural factors not directly measured in the present study. Overall, the findings highlight the potential value of identifying swallowing‐ and feeding‐related difficulties in children with SLD and considering these domains as part of a comprehensive clinical evaluation.

## Declarations

All authors certify that they have no affiliations with or involvement in any organization or entity with any financial interest or non‐financial interest in the subject matter or materials discussed in this manuscript.

## Data Availability

The data used to support the findings of this study are included within the article.
